# Supplementary material for: Experience of irreproducibility as a risk factor for poor mental health in biomedical science doctoral students: A survey and interview-based study
Source: PLoS One. 2023 Nov 15;18(11):e0293584. doi: 10.1371/journal.pone.0293584 (PMC10651026; doi:10.1371/journal.pone.0293584)
Supplement: S1 File — (PDF) [file pone.0293584.s001.pdf]

S1 File: Survey for reproducibility and mental health study

Demographic information

1) Which graduate training program are you supported by?
   a) [list of training programs]

2) How many years have you been enrolled in your current graduate program?
   a) [write in number]

3) Are you of Hispanic, Latino, or Spanish origin?
   a) Yes
   b) No

4) What race do you identify as? (select all that apply)
   a) American Indian or Alaskan Native
   b) Asian
   c) Black or African American
   d) Native Hawaiian or other Pacific Islander
   e) White
   f) Other (please specify) [free text form]

5) What gender do you identify as? (select all that apply)
   a) Man
   b) Woman
   c) Transgender
   d) Nonbinary
   e) Other (please specify) [free text form]

6) Do you come from a disadvantaged background, as defined by the National Institutes of Health? (select all that apply)
   a) I have been or currently am homeless
   b) I have been or currently am in the foster care system
   c) I was eligible for the Federal Free and Reduced Lunch Program for two or more years
   d) I do not have a parent/legal guardian who completed a bachelor's degree
   e) I have been or currently am eligible for Federal Pell grants
   f) I received support from the Special Supplemental Nutrition Program for Women, Infants and Children (WIC) as a parent or child
   g) I grew up in a rural area or a low income/health professional shortage area

Reproducibility experiences

When answering the following questions, please consider all of your scientific experience to date, including lab work in courses, internships, or volunteer positions.

Have you experienced a situation where you were doing an experiment that:

1) Gave you that results were not consistent with what you expected?
   a) I've had one experience like this
   b) I've had several experiences like this
   c) I've never had an experience like this
   d) I'm not sure if I've had an experience like this

2) Had a "right answer," and you did not get that answer?
   a) I've had one experience like this
   b) I've had several experiences like this
   c) I've never had an experience like this
   d) I'm not sure if I've had an experience like this

3) You yourself had done before, but got results that differed from your previous attempts?
   a) I've had one experience like this
   b) I've had several experiences like this
   c) I've never had an experience like this
   d) I'm not sure if I've had an experience like this

4) Someone else in your lab had done before, but you got results that differed from theirs?
   a) I've had one experience like this
   b) I've had several experiences like this
   c) I've never had an experience like this
   d) I'm not sure if I've had an experience like this

5) Was similar to an experiment you'd seen in the published literature, but the results you got differed from what was reported in the publication?
   a) I've had one experience like this
   b) I've had several experiences like this
   c) I've never had an experience like this
   d) I'm not sure if I've had an experience like this

6) Had a control group or comparison group that was supposed to produce an expected result, but you got a different result?
   a) I've had one experience like this
   b) I've had several experiences like this
   c) I've never had an experience like this
   d) I'm not sure if I've had an experience like this

Depression

Over the last 2 weeks, how often have you been bothered by any of the following problems?

1. Little interest or pleasure in doing things
    a. Not at all
    b. Several days
    c. More than half the days
    d. Nearly every day

2. Feeling down, depressed, or hopeless
    a. Not at all
    b. Several days
    c. More than half the days
    d. Nearly every day

3. Trouble falling or staying asleep, or sleeping too much
    a. Not at all
    b. Several days
    c. More than half the days
    d. Nearly every day

4. Feeling tired or having little energy
    a. Not at all
    b. Several days
    c. More than half the days
    d. Nearly every day

5. Poor appetite or overeating
    a. Not at all
    b. Several days
    c. More than half the days
    d. Nearly every day

6. Feeling bad about yourself, or that you are a failure, or have let yourself or your family down
    a. Not at all
    b. Several days
    c. More than half the days
    d. Nearly every day

7. Trouble concentrating on things, such as reading the newspaper or watching television
    a. Not at all
    b. Several days
    c. More than half the days
    d. Nearly every day

8. Moving or speaking so slowly that other people could have noticed. Or the opposite – being so fidgety or restless that you have been moving around a lot more than usual
    a. Not at all
    b. Several days
    c. More than half the days
    d. Nearly every day

Anxiety

Over the last 2 weeks, how often have you been bothered by any of the following problems?

1. Feeling nervous, anxious, or on edge
    a. Not at all
    b. Several days
    c. More than half the days
    d. Nearly every day

2. Not being able to stop or control worrying
    a. Not at all
    b. Several days
    c. More than half the days
    d. Nearly every day

3. Worrying too much about different things
    a. Not at all
    b. Several days
    c. More than half the days
    d. Nearly every day

4. Trouble relaxing
    a. Not at all
    b. Several days
    c. More than half the days
    d. Nearly every day

5. Being so restless that it's hard to sit still
    a. Not at all
    b. Several days
    c. More than half the days
    d. Nearly every day

6. Becoming easily annoyed or irritable
    a. Not at all
    b. Several days
    c. More than half the days
    d. Nearly every day

7. Feeling afraid as if something awful might happen
    a. Not at all
    b. Several days
    c. More than half the days
    d. Nearly every day

General life satisfaction

Indicate how much you agree or disagree with the following statements:

1. My life is going well
    a. Strongly agree
    b. Somewhat agree
    c. Neither agree nor disagree
    d. Somewhat disagree
    e. Strongly disagree

2. My life is just right
    a. Strongly agree
    b. Somewhat agree
    c. Neither agree nor disagree
    d. Somewhat disagree
    e. Strongly disagree

3. I wish I had a different kind of life
    a. Strongly agree
    b. Somewhat agree
    c. Neither agree nor disagree
    d. Somewhat disagree
    e. Strongly disagree

4. I have a good life
    a. Strongly agree
    b. Somewhat agree
    c. Neither agree nor disagree
    d. Somewhat disagree
    e. Strongly disagree

5. I have what I want in life
    a. Strongly agree
    b. Somewhat agree
    c. Neither agree nor disagree
    d. Somewhat disagree
    e. Strongly disagree

End of survey

Thank you for completing the survey!

As a graduate student enrolled at UW Madison, you have access to no-cost, confidential mental health services provided by University Health Services. To get started, you can schedule an access appointment over the phone or using MyUHS. If you need assistance right away, you can call the 24 hour crisis line to speak to an on-call crisis counselor.
