# Supplementary material for: Experience of irreproducibility as a risk factor for poor mental health in biomedical science doctoral students: A survey and interview-based study
Source: PLoS One. 2023 Nov 15;18(11):e0293584. doi: 10.1371/journal.pone.0293584 (PMC10651026; doi:10.1371/journal.pone.0293584)
Supplement: S2 File — (PDF) [file pone.0293584.s002.pdf]

S2 File: Interview guide for reproducibility and mental health study

Background

1. Tell me about the research experiences you had prior to coming to UW Madison.
2. Tell me a bit about the research you're doing now.

Irreproducibility experiences

3. In the survey, you said you'd had one experience/multiple experiences of [survey item(s)]. Can you tell me about the experiences you were thinking of when you answered?
4. Let's focus in on [experience identified in Q3].
    a. Tell me about the steps you took as you were working through that experiment.
    b. Tell me about the process of realizing that you were not going to be able to reproduce the finding/produce a finding.

Expectations and interpretations

5. Did you have any expectations about what the results were supposed to look like before you started the experiment?
    a. Where did you get those expectations from?
6. Why do you think that you were unable to reproduce/produce the results?
    a. Have your thoughts about the source of the problem changed over time?
    b. Do you think that getting results in your field depends strongly on individual skill or having great "bench hands"?

Help seeking behavior

7. Did you talk to anyone about the difficulties you were experiencing with the experiment?
    a. How long was it from when you first started experiencing these problems to when you reached out to someone?
    b. What kind of explanations/advice did they offer?
    c. Did you talk to your PI/advisor specifically?

Correcting the research record

8. What happened to the data from your experiments that failed to replicate/produced null results?
    a. Did you try to publish these findings?
    b. Did you abandon this line of research?

9. Repeat Q4-Q8 for a second experience, if appropriate.

Reproducibility training

10. Was the "reproducibility crisis" a topic of conversation in your lab/research group or coursework?
    a. What do you remember from these classes/conversations?
    b. Did these conversations have an impact on your thinking about your own experiences with irreproducibility/null findings?

Impact

11. What impact, if any, did these failure(s) to replicate/null finding(s) have on you?
12. Do you think these experiences had an impact on your:
    a. Progress through your program or your career trajectory?
    b. Enthusiasm for science?
    c. Mental health?

Wrap-up

13. Is there anything else about these experiences you'd like to tell me that my questions didn't capture?
14. Would you like information on how to access mental health services at UHS? Would you like help booking an access appointment at UHS to get started?
