# Supplementary material for: Experience of irreproducibility as a risk factor for poor mental health in biomedical science doctoral students: A survey and interview-based study
Source: PLoS One. 2023 Nov 15;18(11):e0293584. doi: 10.1371/journal.pone.0293584 (PMC10651026; doi:10.1371/journal.pone.0293584)
Supplement: S3 File — (PDF) [file pone.0293584.s003.pdf]

S3 File: Code book for analysis of interview data from reproducibility and mental health study

Variable	Value	Explanation
ID		A unique identifier assigned to each study participant
Experience	1	The first experience described by the participant
	2	The second experience described by the participant
Type of irreproducibility (Q5a)	Someone else's work	Prior results were obtained by someone the participant had a social relationship with (e.g., current or former lab mate, collaborator from another lab)
	Literature/prior hypothesis	Prior results or hypothesis about expected results were derived from the published literature
	Own work	Prior results were obtained by the participant
Time period (Q3)	Pre grad school	Experience took place during undergrad or pre-doctoral training
	During grad school	Experience took place during MA or PhD work (including graduate work outside of UW Madison)
Most prominent emotional response (Q11)	Panic/anxiety	Participant described their experience in terms of panic or anxiety
	Self-doubt/loss of confidence	Participant described their experience in terms of self-doubt or loss of confidence in their scientific abilities
	Depressed/demotivated/tired	Participant described their experience in terms of depression, lack of motivation, or feeling tired/exhausted because of the experience
	Annoyed/ frustrated	Participant described their experience in terms of annoyance or frustration
	Worry about wasting time/ resources	Participant described their experience in terms of worry about wasting their time, other's time, or resources (NOTE: this code was collapsed into panic/anxiety in the final analysis)
	Indifference/lack of surprise	Participant did not describe any strong emotional response to the experience or described themselves as not being surprised by the anomalous results
Did the response interfere with daily activities? (Q11, 12c)	Y	Participant mentioned disruptions to sleep, eating, ability to focus inside or outside of work, etc., resulting from their response to the experience
	N	Participant did not mention any disruptions to daily activities
Most prominent attribution (Q6)	Own fault	Participant attributed results to an objective error they made or to their lack of knowledge or skill
	Bad reagents/ equipment	Participant attributed results to an issue with reagents or equipment used (e.g., a spoiled buffer or unspecific binding from an antibody)

	Problem with protocol	Participant attributed results to problem with the protocol (e.g., lacking sufficient information about the original protocol or needing to “dial in” their protocol)
	Difference between original and new experiment	Participant attributed results to differences between original experiment and their experiment (e.g., minerals in the water used, species differences)
	Tricky/complex phenomena being studied	Participant attributed anomalous results to complex nature of what was being studied
	Original/result hypothesis is wrong	Participant described their results as correct and the original hypothesis/result as incorrect
Initial attribution [if mentioned] (Q6a)	Own fault	Participant initially attributed results to something they did or their abilities, but ultimately attributed outcome to something else
	Bad reagents/ equipment	Participant initially attributed results to an issue with reagents or equipment used, but ultimately attributed outcome to something else
	Problem with protocol	Participant initially attributed results to problem with the protocol, but ultimately attributed the outcome to something else
	Difference between original and new experiment	Participant initially attributed results to differences between original experiment and their experiment, but ultimately attributed the outcome to something else
	Tricky/complex phenomena being studied	Participant initially thought the outcome was not error and due to complex nature of what is being studied, but ultimately attributed the outcome to something else
	Original/result hypothesis is wrong	Participant initially thought outcome was not error and the original result was incorrect, but ultimately attributed the outcome to something else
	Not mentioned	Participant did not mention initial attribution, only their attribution at the time of the interview
Initial help seeking behavior (Q7, 7c)	Try to fix on own	Participant’s first response to the results was to try to fix the anomalous results on their own
	Go to PI	Participant’s first response to the results was to go to their PI for help
	Go to peers	Participant’s first response to the results was to consult other graduate students, other researchers, or someone else in the lab (not PI) for help
Comparison to peers	Normalizing	Participant compared their experiences to peers who had similar experiences, normalizing the experience of irreproducibility
	Feeling worse	Participant compared their experience to peers who appeared to be having more success and it made them feel worse about themselves or their abilities

Eventual outcome (Q8b)	Solved problem	Participant solved the problem and produced desired results, or arrived an explanation for why they would not be able to produce the desired results
	Abandon research question	Participant abandoned their original research question
	Shift research question	Participant altered their original research question
Was the data published? (Q8a/b)	Y	Data from the experience were published or are currently being written up
	N	Data from the experience were not published
Agency	0	Participant describes feeling little or no control over the experimental results or decisions about whether to move forward with or abandon line of research
	1	Participant makes no positive or negative comments about agency
	2	Participant makes decisions about continuing or abandoning research rather than PI, describes feeling empowered to solve their own problems
Access to PI (Q7c)	0	Participant mentions difficulties in meeting with or getting a response from PI
	1	Participant makes no positive or negative comments about access to PI
	2	Participants mentions regular meetings with PI or an "open door" policy
Relationship with PI	0	Participant speaks negatively about PI's advice, training, or mentorship, or feels blamed or criticized by the PI
	1	Participant provides neutral or no descriptions of PI
	2	Participant praises PI's advice, training, or mentorship, or feels supported by the PI
Lab culture	0	Participant speaks negatively about the lab dynamic, describing a culture of pressure, overwork, or competition
	1	Participant provides neutral or no descriptions of the lab
	2	Participant praises the lab culture, feels supported by others in their lab or related labs, describes a collaborative environment
Awareness of reproducibility (Q10a/b)	0	Participant describes themselves as unaware of reproducibility issues
	1	Participant describes hearing about reproducibility issues in coursework or in passing in classes/lab meetings
	2	Participant describes themselves as very aware of reproducibility issues, mentions reading about the issue independently of coursework, participating in conversations about reproducibility and rigor, etc.
Overall impact (Q12)	0	Participant believes the experience negatively impacted their career trajectory, mental health, and/or enthusiasm for science

	1	Participant believes the experience had no strong impact on their career trajectory, mental health, and/or enthusiasm for science
	2	Participant believes the experience has a positive impact on their career trajectory, mental health, and/or enthusiasm for science
**Did the participant mention:**		
Field norms	Y	Participant describes norms around whether null/conflicting results are considered interesting or publishable in their field
	N	Participant does not describe their field's norms around irreproducibility
External support networks? (Q7)	Y	Participant describes receiving support from people or services outside of the lab
	N	Participant did not mention support from people or services outside of the lab
A label for their mental health struggle? (Q12c)	Y	Participant labelled mental health problems they faced due to their experience (e.g., anxiety, depression, suicidal ideation)
	N	Participant did not label a specific mental health problem
Seeking mental health treatment?	Y	Participant mentioned seeking mental health treatment due to their experience
	N	Participant did not mention seeking mental health treatment due to their experience
Success in other projects? (Q1)	Y	Participant mentioned the importance of having success in other research projects to their feelings of competence or their PI's appraisal of the situation
	N	Participant did not mention success in other research projects or experiments
Changing their reporting practices?	Y	Participant mentioned changing their reporting practices due to their experience (e.g., writing more detailed methods sections)
	N	Participant did not mention changes to their reporting practices
Changing their experimental practices?	Y	Participant mentioned changing their experimental practices due to their experience
	N	Participants did not mention changing their experimental practices due to their experience
Become more skeptical of the literature?	Y	Participant mentioned becoming more skeptical of scientific literature after their experience
	N	Participant did not mention becoming more skeptical of scientific literature after their experience
