# Supplementary material for: Experience of irreproducibility as a risk factor for poor mental health in biomedical science doctoral students: A survey and interview-based study
Source: PLoS One. 2023 Nov 15;18(11):e0293584. doi: 10.1371/journal.pone.0293584 (PMC10651026; doi:10.1371/journal.pone.0293584)
Supplement: Attachment — Submitted filename: PLOS_ONE_reviewer_response.docx [file pone.0293584.s005.docx]

**PONE-D-22-24719R1 response to reviewers**

**Editor**

1. **Ensure that the manuscript meets PLOS ONE requirements for file naming**.
   We are guessing that this request refers to the citations rather than the file names—we did not find any errors in the file names, but we did find that our citations had not been generated using the proper style. We have redone the citations/bibliography and removed the Zotero field codes to prevent potential problems after uploading. Additionally, we have checked the image files using PACE and changed the names of the supplementary files to reflect the addition of one new file.
2. **Remove Bewick et al from reference list because it is unpublished.**
   There is no reference to a paper by Bewick et al in our manuscript.
3. **Review reference list and justify citations to retracted papers.**
   As far as we are aware, there are no citations to retracted papers in our manuscript. If the editor is aware of issues with some of the papers cited, please let us know which papers and we will happily revise the manuscript.

**Reviewer One**

1. **Give examples of the survey questions.**
   We have added a new supplementary file (S1 File) that contains the survey in its entirety.
2. **Update the literature review.**
   We have used several recently published systematic reviews/meta-analyses/meta-syntheses to more comprehensively describe the relevant literatures, particularly the literature on graduate student mental health, which the reviewer rightly notes has been very active in the past few years (likely owing to the mental health impacts of the Covid-19 pandemic).
3. **Perform additional quantitative analyses, such as correlations or linear regressions. Specifically, perform correlation/multivariate regressions on the mental health measures and questions about irreproducibility in the survey data.**
   We have elected not to perform these analyses because we don’t believe that looking for relationships between current mental health and past experiences of reproducibility would be appropriate given the data we have collected. We did not collect data describing when participants experienced irreproducibility, only their lifetime to date experiences of irreproducibility. If we were to perform the analyses suggested, we would be comparing irreproducibility events with very different recency to current mental health with no way of controlling for time elapsed. We collected current mental health data primarily for the purposes of comparing our population to reference populations to assess whether we had a significantly lower/higher frequency of current mental illness in our population. We have added additional detail about this rationale in the new section in the materials and methods described below.
4. **Describe the analysis of the survey data in the materials and methods section.**
   A new section on the analysis of the survey data has been added to the materials and methods section.

**Reviewer Two**

1. **Describe the potential impact of the pandemic on the study.**
   We now describe two potential impacts of the pandemic on the study: we may have experienced a high response rate due to participants’ inability to access their lab spaces (and therefore having fewer work tasks to complete), and we may have experienced a lower response rate from women because of women’s increased participation in caretaking labor during the pandemic. We also clarify that the pandemic almost certainly had an impact on participants’ mental health, and therefore it is particularly important that we are comparing current mental health data from our population to data taken from the HMS survey during the same time period.
2. **Account for possible response bias in the study.**
   We now clarify in the methods section that the reason for comparing our population to the HMS study population is because the HMS survey had complete data on the populations sampled and was therefore able to control for response bias. We did not have access to data for non-responders and therefore could not make corrections for response bias, but we assume that similar response biases are operative in our study as were seen in the HMS study. We now show both the raw and weighted data from the HMS survey in Table 1 to make those biases clear, and explicitly discuss response bias in the manuscript.
3. **Explain why the percentage of women who participated in the study was lower.**
   See the response to question R2.1 above
4. **Describe the time needed to answer the survey, and any internal consistency tests performed to verify that the quality of the responses was not impacted by answering three surveys simultaneously.**
   We have added information on the survey duration to the methods section. We did not perform any internal consistency tests because the total time taken to complete the survey was less than six minutes. As mentioned above, we have also now included the survey as an appendix so that readers can get a better sense of the total length of the survey.
5. **Clarify that the HMS study data was used for external validation in the methods section**.
   We have clarified this in the methods section.
6. **Describe the statistical analyses used to evaluate the quantitative data in the methods section.**
   We have added a new section to the materials and methods, as also suggested by R1 above.
7. **Clarify that the coding was performed by the same researchers who took part in the interviews.**
   We have clarified this in the qualitative data analysis methods section and clarified that the interview transcripts were identified by participant ID only during the qualitative data analysis phase.
8. **Variation in how participants respond to/interpret failures to replicate may be related to personality traits or prior levels of self-efficacy.**
   We think this is an excellent suggestion for future research and have added it to the discussion section.
9. **Consider whether indifference is better interpreted as apathy or as resilience to the experience of failing to replicate a result.**
   We thank the reviewer for this helpful comment—we do not think that what we initially classified as “indifference” is best interpreted as apathy, and so have changed the descriptor for this category to “equanimity.” We believe this better captures the spirit of this category and helps avoid misreading this category of emotional response as apathy or anhedonia, which would fall under the category we have termed “depressed/demotivated/tired.”
10. **Clarify whether the interview excerpts in Box 1 come from different participants**.
    Each quote in Box 1 comes from a different participant. We have added participant ID numbers to all interview excerpts throughout the manuscript to clarify this.
11. **Add a concluding paragraph to the discussion.**
    We have added a concluding paragraph.
12. **Adjust the color scheme in Figure 1.**

We have changed the color palette from viridis to scico, which is another scientifically-derived color map that has been designed for perceptual uniformity, to be interpretable by readers with different forms of color blindness, and to render properly in greyscale.
